# Hit the acceleration petal: Methylation as a mediator of ethylene-induced petal senescence

**DOI:** 10.1093/plphys/kiad116

**Published:** 2023-02-22

**Authors:** Aida Maric

**Affiliations:** Assistant Features Editor, Plant Physiology, American Society of Plant Biologists, USA; CIBSS—Centre for Integrative Biological Signalling Studies, University of Freiburg, Habsburgerstraße 49, 79104, Freiburg, Germany; Plant Environmental Signalling and Development, Institute of Biology III, University of Freiburg, Schänzlestraße 1, 79104 Freiburg, Germany

Carnation (*Dianthus caryophyllus* L.) flowers probably originated in the Mediterranean region and certainly were known by the ancient Greeks; the word *dianthus* is attributed to Greek botanist Theophrastus and refers to *dios* (divine) and *anthos* (flower). Since ancient times and through the middle ages, carnations have appeared in art, literature, and ceremonies to become a part of modern pop culture. Today, carnations are economically one of the most important cut flowers (Li et al. 2019). Therefore, understanding the processes regulating their shelf life is of great interest to the growing cut flower industry. The longevity of this floricultural crop is largely determined by petal senescence (Ma et al. 2018). Petal senescence is a tightly regulated active developmental process promoted by the plant hormones ethylene and abscisic acid (Ma et al. 2018). Carnations are highly sensitive to ethylene, a gaseous phytohormone that regulates different aspects of plant life and development (Sasidharan and Voesenek 2015; Maric and Hartman 2022).

Post-translational modifications of histone tails create epigenetic signatures that lead to changes in gene expression. These histone modifications come in the form of different covalent groups whose number and deposition region affect gene expression. Deposition of the trimethyl group at the lysine 4 residue of histone 3 (H3K4me3) is a well-known mark traditionally connected to transcriptional activation (Foroozani et al. 2021). Histone modifications are highly dynamic mechanisms controlling plant development and stress response (Foroozani et al. 2021). For example, H3K4 methylation mediates senescence processes (Brusslan et al. 2015). Additionally, ethylene controls a variety of plant responses at the epigenetic level (Zhang et al. 2016). However, the way specific epigenetic factors regulate ethylene-mediated petal senescence remains elusive.

**Figure 1.**
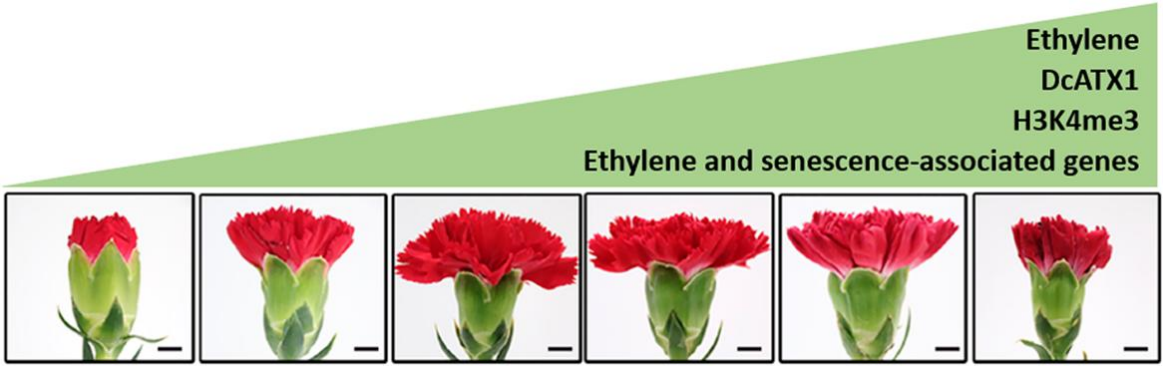
Carnation flower from budding phase to senescence. Petal senescence is induced by increasing levels of ethylene. Moreover, ethylene can induce DcATX1 protein levels, thereby increasing H3K4me3 deposition at the promoters of target genes. These targets are ethylene biosynthesis- and senescence-associated genes accelerating petal senescence. Scale bar = 1 cm. Modified from Feng et al. (2023).

In this issue of *Plant Physiology*, Feng et al. (2023) used a combination of genetic and molecular approaches to investigate the intricate relationship between ethylene and histone modifications driving petal senescence in carnation. Using total protein extracts and antibodies directed against H3 and H3K4me3, the authors examined the relative amount of H3K4me3 modification over time. As expected, ethylene treatment at the budding stage of flower development accelerated petal senescence but also led to H3K4me3 accumulation (Fig. 1).

The authors showed that the carnation homolog of ARABIDOPSIS HOMOLOG OF TRITHORAX1 (ATX1) protein—DcATX1—is stabilized by ethylene and throughout senescence. By combining the genetic approach and ethylene treatment experiments, the authors showed that overexpression of *DcATX1* promotes ethylene-controlled petal senescence while silencing of *DcATX1* postpones it. According to the eFP database, compared with other members of the same methyltransferase family, Arabidopsis (*Arabidopsis thaliana*) *ATX1* has higher expression levels in petals during later stages of flower development (Winter et al. 2007; Feng et al. 2023). Using carnation *DcATX1* mis-expression lines, the authors showed a positive association between H3K4me3 accumulation and *DcATX1* levels; i.e. *DcATX1* over-expression plants had higher levels of the H3K4me3 mark while *DcATX1* silenced plants accumulated less H3K4me3. Together with results of an in vitro methyltransferase assay showing that DcATX1 itself is enough to catalyze methylation at H3 and accumulate H3K4me3, Feng et al. (2023) confirmed that DcATX1 acts as a methyltransferase in carnation plants and is involved in ethylene-mediated petal senescence.

In an attempt to identify downstream targets of DcATX1-mediated methylation changes, Feng et al. (2023) turned to a target group of ethylene- and senescence-induced genes. Using chromatin immunoprecipitation-qPCR (ChIP-qPCR) experiments, the authors immunoprecipitated H3K4me3 and identified ethylene-induced H3K4me3 enrichment in promoters of the selected genes. Based on the role of DcATX1 as a methyltransferase, authors used a combination of reverse transcription quantitative PCR (RT-qPCR) and ChIP-qPCR to show that DcATX1 regulates the expression of target genes by regulating H3K4me3 levels. Furthermore, ChIP-qPCR using a DcATX1-specific antibody was performed to show that ethylene-treated carnation petals show higher levels of DcATX1 binding in the promoters of target genes.

Research connecting ethylene signaling and epigenetics opens space for novel insight into traditionally well-documented developmental mechanisms like petal senescence. Taken together, the elegant experiments reported by Feng et al. (2023) give us interesting information about the H3K4me3 accumulation dynamics during different stages of flower opening and the senescence process (Fig. 1). In light of a report showing an ethylene-mediated increase in activating epigenetic marks—H3K14 and H3K23 acetylation—it could be interesting to study the accumulation dynamics of these two marks in carnation flowers (Zhang et al. 2016). Do they work together with the H3K4me3 activation mark? Or do they have different target genes? The same report shows that an important ethylene signaling protein, ETHYLENE INSENSTIVE2 (EIN2), mediates ethylene-induced acetylation. The results from Feng et al. (2023) indicate that ethylene contributes to DcATX1 protein stability. However, the exact mechanism of DcATX1 stabilization through ethylene has not been explored in mechanistic detail. It would be interesting to see if EIN2 participates in the regulation of DcATX1 to control the accumulation of the H3K4me3 mark. Combined, these further experiments could tell us how specific is the ethylene-induced senescence. We are for sure on a very exciting road to understanding ethylene-regulated epigenetic marks to ameliorate the longevity of economically important cut flowers.
